# Integral Whole Brain Dose from Stereotactic Radiosurgery of 47 Metastatic Lesions: A Dosimetric Case Study

**DOI:** 10.7759/cureus.436

**Published:** 2015-12-27

**Authors:** Javad Rahimian, Joseph C Chen, Michael J Miller, Kenneth Lodin, Michael R Girvigian

**Affiliations:** 1 Radiation Oncology, Southern California Permanente Medical Group; 2 Neurosurgery, Southern California Permanente Medical Group

**Keywords:** Stereotactic Radiosurgery, whole brain irradiation, dosimetry, polymetastases, alk-positive adenocarcinoma

## Abstract

This report describes the case of a 15-year-old male diagnosed with primary ALK-positive adenocarcinoma of the lung metastatic to the brain. He was treated with surgical resection for a single lesion followed by whole brain radiotherapy and subsequently underwent 10 courses of stereotactic radiosurgery for 47 lesions delivered over a four-year period. Currently, all metastatic lesions in the brain are completely resolved or locally controlled.

## Introduction

The spread of systemic cancer to the brain is a common complication that occurs in up to 40% of all patients and is generally associated with a poor prognosis [1]. The most common route of metastatic dissemination resulting in brain metastases is hematogenous. It is presumed that the entire brain is “seeded” with micrometastatic disease, even when only a single intracranial lesion is detected. Therefore, whole-brain radiation therapy (WBRT) had been recommended and is a mainstay of treatment [2]. Patients treated with stereotactic radiosurgery (SRS), plus WBRT, however, are at a greater risk of a significant decline in learning and memory function at four months, compared with groups receiving SRS alone [3-4]. Improvements in systemic therapies have also increased long-term survival and put a greater number of patients at risk for late complications of WBRT.

Stereotactic radiosurgery, a technique that permits the precise delivery of a high-dose of radiation to a small intracranial target while sparing the surrounding normal brain, is becoming an increasingly preferred treatment for brain metastases. Radiosurgery is an effective, minimally invasive outpatient treatment option for small intracranial metastases. It not only provides local control rates equivalent to these from surgical series, but also is effective in treating patients with surgically inaccessible lesions, with multiple lesions, or with tumor types that are resistant to conventional treatments [5]. Currently, we are able to deliver high doses to lesions in the brain with high precision using frameless image-guided radiosurgery [6].

As adverse radiation effects, including neurocognitive function, are dose-dependent, we intend to determine and compare the integral dose received by one patient treated with both one course of whole brain radiation as well as 10 courses of stereotactic radiosurgery for 47 subsequent lesions.

The purpose of this study is to determine the integral whole brain dose received from both the whole brain radiotherapy followed by stereotactic radiosurgery of 47 lesions over a four-year period in a 15-year-old patient.

## Case presentation

A 15-year-old Latino male with no family history of lung cancer was diagnosed with primary ALK-positive adenocarcinoma of the lung (diagnosed in August 2011). Upon presentation, the patient was found to have multiple metastases to the brain and was treated with surgical resection for a single posterior fossa lesion followed by whole brain radiation. Multiple recurrences of the disease were followed by 10 courses of SRS for 47 metastatic lesions. Whole brain radiotherapy was delivered with opposed lateral beams and consisted of 35 Gy in 14 fractions. Linear accelerator-based SRS was performed via multi-isocenter techniques, using 4-5 cone-based circular arcs per lesion, except for two lesions that were treated with five dynamic conformal arcs. The patient was immobilized using a bivalve mask for frameless image-guided radiosurgery using a Novalis linear accelerator. The median dose to the peripheral margin of the lesions was 20 Gy. The whole brain tissue, minus the volume of SRS-treated lesions, was contoured. The integral dose (Joules or Gy-Kg) to the normal whole brain tissue was then calculated using the dose volume histograms (DVH) for both the whole brain radiotherapy and the SRS treatments. This was divided by the mass of normal brain in Kg to give the units of Gy. All planning was done in the BrainLab 4.5.2 with SRS performed on the Novalis linear accelerator with ExacTrac^©^ 6D Robotic and stereoscopic image guidance.

Table 1 summarizes the dates, the number of lesions, lesion size, and the calculated integral dose to the whole brain received from the WBRT as well as 10 courses of SRS treatments tabulated both in Gy and Joules.

**Table 1:** A summary of lesions' characteristics and dosimetry for one course of WBRT and 10 courses of SRS.

COURSE NO.	TREATMENT DATE	NO. OF LESIONS	MEAN LESION VOL.	VOLUME RANGE	TOTAL LESIONS VOL.	WHOLE BRAIN DOSE	WHOLE BRAIN DOSE	ISOCENTER DOSE
			(cc)	(cc)	(cc)	(Joules)	(Gy)	(Gy)
1	August-11	WHOLE BRAIN	N/A	N/A	N/A	65.125	36.041	35
2	January-13	1	0.529	0.529	0.529	1.292	0.697	25
3	August-13	13	0.224 ± 0.172	0.647 - 0.037	2.917	4.006	2.376	20
4	December-13	3	0.088 ± 0.048	0.125 - 0.034	0.265	0.798	0.464	20
5	February-14	5	0.0526 ± 0.0264	0.026 - 0.097	0.263	1.198	0.263	20
6	May-14	4	0.185 ± 0.118	0.083 - 0.302	0.740	1.686	0.960	22.5-25
7	July-14	2	0.117 ± 0.093	0.051 - 0.182	0.233	0.576	0.333	25
8	September-14	9	0.121 ± 0.100	0.026 - 0.292	1.089	2.339	1.409	19.14-25
9	November-14	1	0.107	0.107	0.107	0.230	0.138	25
10	February-15	5	2.471 ± 4.586	0.035 - 10.602	12.254	4.431	2.593	22.5-25
11	October-15	4	0.0265 ± 0.0205	0.013 - 0.057	0.106	0.871	0.519	25
	Total SRS:	47		0.013-10.602	18.503	17.427	9.752	

**Figure 1:**
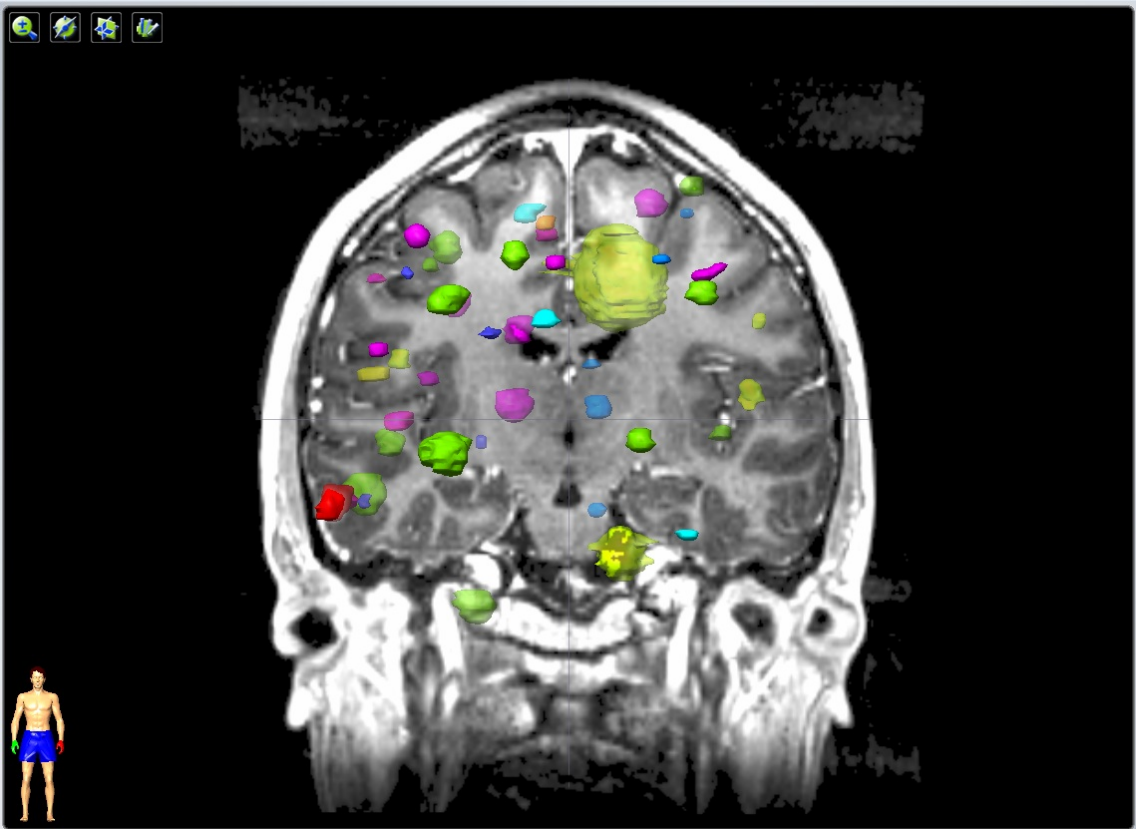
The treated lesions superimposed on pre-SRS MRI scan Forty-seven lesions superimposed on a coronal MRI image plane scanned prior to the first course of SRS. The lesions are color-coded; each color represents the lesions treated in the same course of stereotactic radiosurgery.

Figure 1 shows the 47 metastatic lesions superimposed on the MRI coronal plane imaged for the first SRS treatment. The lesions are color-coded; each color represents the lesions treated in the same course of stereotactic radiosurgery.

**Figure 2:**
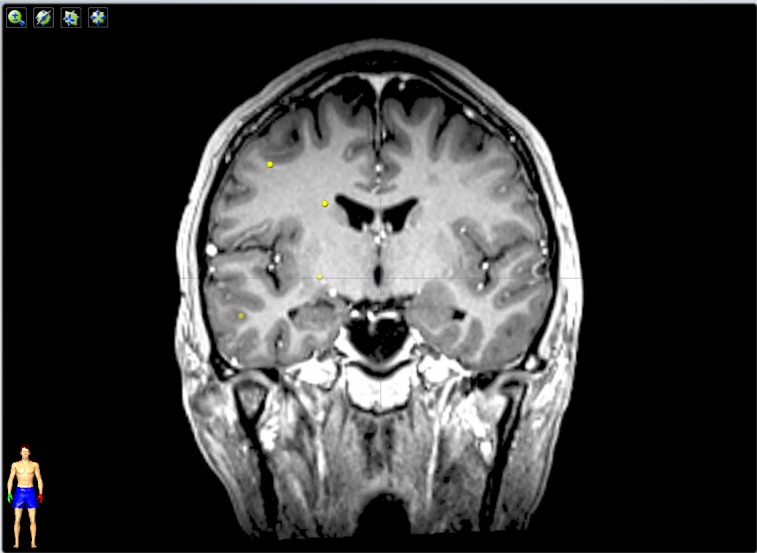
Post-SRS MRI scan The latest MRI of the brain shows complete resolution and local control of the treated lesions.

Figure 2 is the post-treatment MRI showing complete resolution of all the treated oligometastatic lesions and local control of the two larger lesions.

The patient has been treated with 18 courses of 250 mg of crizotinib twice a day from October 2011 to July 2014 followed by 150 mg of ceritinib (Zykadia) four times a day from October 2014 to the present time.

Informed patient consent was obtained for each treatment.

## Discussion

The integral radiation dose contributed to the normal whole brain tissue from the 10 courses of SRS treatments is calculated to be 9.752 Gy, significantly lower than the dose received from whole brain radiotherapy (36.041 Gy). The patient tolerated the treatments without significant adverse effects. The patient is attending college and has developed moderate neurocognitive deficit affecting his academic achievement. This may be likely due to upfront whole brain radiotherapy, chemotherapy, and cumulative brain injury from polymetastases.

It has been suggested that radiosurgery without prophylactic WBRT could be a primary choice of treatment for patients with as many as 10 cerebral metastases from non-small cell cancer. Stereotactic radiosurgery safely and effectively treats intracranial disease with a high rate of local control in patients with 10 or more brain metastases. In patients with fewer metastases, a non-melanoma primary lesion, controlled systemic disease, and a low RPA class, SRS may be most valuable. In selected patients, it can be considered as first-line treatment [7].

## Conclusions

SRS treatment of multiple brain metastases delivers a significantly less integral radiation dose to normal brain tissue than whole brain radiotherapy. With multi-isocenter linear accelerator based treatments, multiple small lesions can be treated to a high dose while still sparing significant normal brain tissue. This dosimetric advantage likely has clinical benefit for late neurocognitive preservation. SRS of multiple metastatic lesions should be considered over whole brain radiotherapy in patients where minimizing integral brain dose and late neurocognitive deficit is indicated. SRS can be used in effective control for polymetastases.
